# Knowledge mapping of UMOD of English published work from 1985 to 2022: a bibliometric analysis

**DOI:** 10.1007/s11255-023-03664-4

**Published:** 2023-06-15

**Authors:** Guannan Sun, Chao Liu, Chengcheng Song, Xiaodong Geng, Kun Chi, Zhangning Fu, Quan Hong, Di Wu

**Affiliations:** 1 Medical School of Chinese PLA, Beijing, 100853 China; 2 State Key Laboratory of Kidney Diseases, Department of Nephrology, First Medical Center of Chinese, National Clinical Research Center for Kidney Diseases, PLA General Hospital, Beijing, 100853 China; 3 Department of Nephrology, Beijing Electric Power Hospital, Beijing, 100073 China

**Keywords:** UMOD, Uromodulin, Bibliometric analysis, CKD

## Abstract

**Background:**

UMOD is exclusively produced by renal epithelial cells. Recent genome-wide association studies (GWAS) suggested that common variants in UMOD gene are closely connected with the risk of CKD. However, a comprehensive and objective report on the current status of UMOD research is lacking. Therefore, we aim to conduct a bibliometric analysis to quantify and identify the status quo and trending issues of UMOD research in the past.

**Methods:**

We collected data from the Web of Science Core Collection database and used the Online Analysis Platform of Literature Metrology, the Online Analysis Platform of Literature Metrology and Microsoft Excel 2019 to perform bibliometricanalysis and visualization.

**Results:**

Based on the data from the WoSCC database from 1985 to 2022, a total of 353 UMOD articles were published in 193 academic journals by 2346 authors from 50 different countries/regions and 396 institutions. The United States published the most papers. Professor Devuyst O from University of Zurich not only published the greatest number of UMOD-related papers but also is among the top 10 co-cited authors. KIDNEY INTERNATIONAL published the most necroptosis studies, and it was also the most cited journal. High-frequency keywords mainly included ‘chronic kidney disease’, ‘Tamm Horsfall protein’ and ‘mutation’.

**Conclusions:**

The number of UMOD-related articles has steadily increased over the past decades Current UMOD studies focused on Biological relevance of the UMOD to kidney function and potential applications in the risk of CKD mechanisms, these might provide ideas for further research in the UMOD field.

## Introduction

Tamm–Horsfall protein is the most abundant urinary protein in physiological conditions, and was discovered by Igor Tamm and Frank Horsfall in 1950 [1]. Muchmore and Decker isolated a glycoprotein with immunomodulatory activity, which was named uromodulin (UMOD), from the urine of pregnant women In 1985 [2]. However, in 1987, it is demonstrated that Tamm–Horsfall protein and UMOD were, in fact, the same protein product [3]. UMOD is mostly (90%) produced by epithelial cells lining the thick ascending limb of Henle’s loop and to a lesser extent (10%) by epithelial cells of the early part of the distal convoluted tubule [4]. In the past two decades, Interest in UMOD was boosted by genetic studies that reported involvement of the UMOD gene, which encodes UMOD, in a spectrum of rare and common kidney diseases. This protein might regulate salt transport, protect against urinary tract infection and kidney stones, and have roles in kidney injury and innate immunity [5]. A recent meta-GWAS showed that UMOD is among the most outstanding loci associated with CKD in the general population, and it has a large effect on eGFR and CKD risk, a condition that affects up to 10% of the population worldwide [6]. In recent years, a positive correlation between serum uromodulin levels and kidney function was confirmed by several studies on populations with kidney disease of different etiology [7]. Moreover, serum UMOD was shown to be more sensitive than conventional markers (serum creatinine, urea, and cystatin C) reflecting glomerular filtration to detect early stage CKD [8]. A lately prospective cohort study enrolled 1538 hospitalized patients in a multicenter reminded that higher urinary uromodulin levels were associated with smaller eGFR declines and decreased incidence of the composite kidney outcome [5]. These findings raise the possibility that modulating circulating UMOD levels could have therapeutic potential on CKD. Therefore, we undertook a bibliometric analysis and visual analysis to explore the hotspots and frontier directions to gain further insight into the pathways connecting UMOD and CKD, hoping to provide researchers with some useful guidance.

## Materials and methods

### Data collection

The Web of Science Core Collection (WoSCC) database is widely used in bibliometrics, which contains Science Citation Index Expanded (SCIE),Social Science Citation Index (SSCI), and Emerging Sources Citation Index (ESCI) [9, 10]. The data search was conducted on 5 July 2022 through the WoSCC database from 1985 to 2022. The search formula was [TS = (Tamm–Horsfall protein) OR TS = (UMOD) OR TS = (umod) OR TS = (thp)] AND [TS = (chronic kidney disease) OR TS = (chronic renal disease)]. Search results were downloaded as “Full Record and Cited References”.

### Data analysis

Cite Space 6.1.R2, a bibliometric and visual analysis tool that excels at detecting cooperation, key points, internal structure, potential trends, and dynamics in a scientist field [11]. The Online Analysis Platform of Literature Metrology and Microsoft Excel 2019 were used to perform bibliometric analysis and visualization. The retrieved data was collected within 1 day to avoid any potential deviation due to the daily updating of the database. Total of 353 papers were obtained. Bibliographic information was collected, including the year of publication, authorship, publishing journals, institution, country of origin and keywords. Furthermore, the impact factor (IF) and Journal citation reports (JCR) division of journals and the H-index of scholars were obtained from the Web of Science on January 5, 2023.

## Results

### Overview of publications on UMOD

**Fig. 1:**
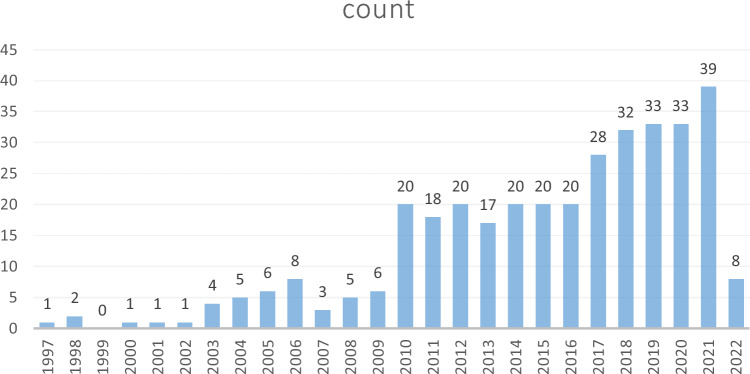
Number of publications by year from 1997 to 2022

**Fig. 2:**
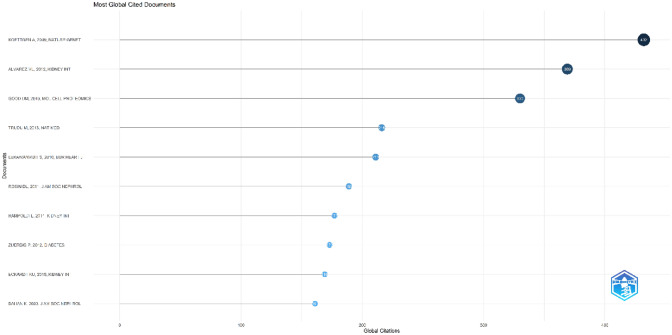
Most global cited documents

Figure 1 shows the annual number of UMOD-related publications which continually increased over time. The annual growth rate is 8.67%. More scholars started to research in this field especially after the year of 2010. The number of UMOD-related articles has steadily increased over the past decade. A Johns Hopkins University research team conducted genome-wide association studies to identify susceptibility loci for chronic kidney disease, which found that rare mutations in UMOD may influence renal function and cause mendelian forms of kidney disease [12], which caused widely attention in this field, and it was cited by 432 times (Fig. 2). This high index article may contribute to the continually increased researches in this field since the year of 2010.

### Distribution characteristics of the literature

This study design contains articles (*n* = 281), review articles (*n* = 58), meeting abstract (*n* = 7), editorial materials (*n* = 6), and proceeding paper (*n* = 1) (Table 1). The most cited article was published in 2009 with the citation being 432 [12]. M Lucrecia Alvarez’s research published in 2012 was cited 369 times [13] (Fig. 2). The oldest paper on the list was published in 1997 by B Settergren et al. and was cited 23 times [14]. The most recent paper has not been cited and was published in 2022 by Laura et al. [15]. Moreover, 20 documents are cited over 100 times.

**Table 1:** Document types

Ranking	Type	Counts (%)
1	Article	281
2	Review	58
3	Meeting abstract	7
4	Editorial material	6
5	Proceeding paper	1

### Distribution characteristics of countries/regions and institutions

**Fig. 3:**
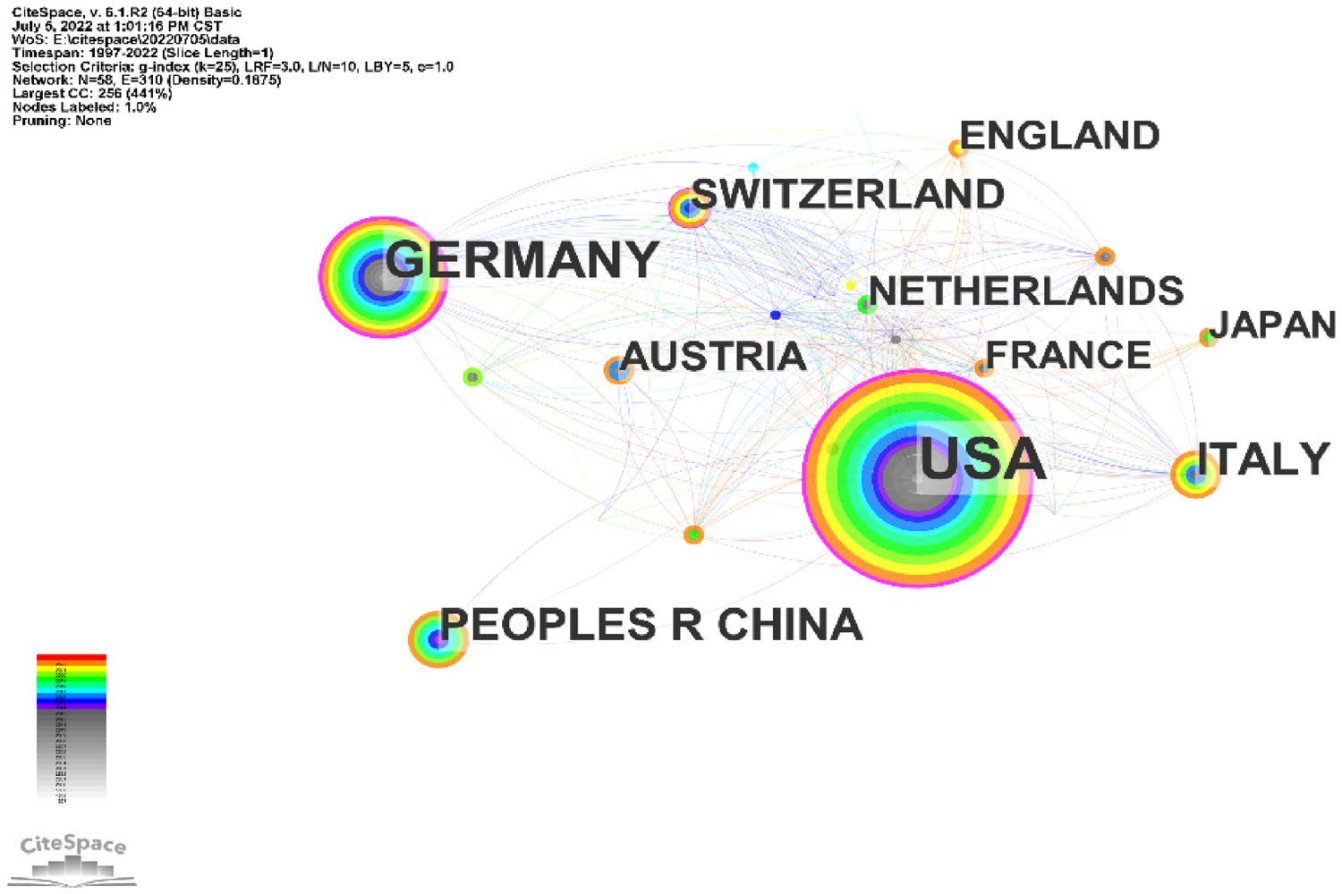
Co-occurrence map of countries. The node size reflects the co-occurrence frequencies, and the links indicate the co-occurrence relationships. The color of node and line represent different years; colors vary from purple to red as time goes from 1997 to 2022, and nodes with purple round mean high betweenness centrality (≥ 0.1)

**Fig. 4:**
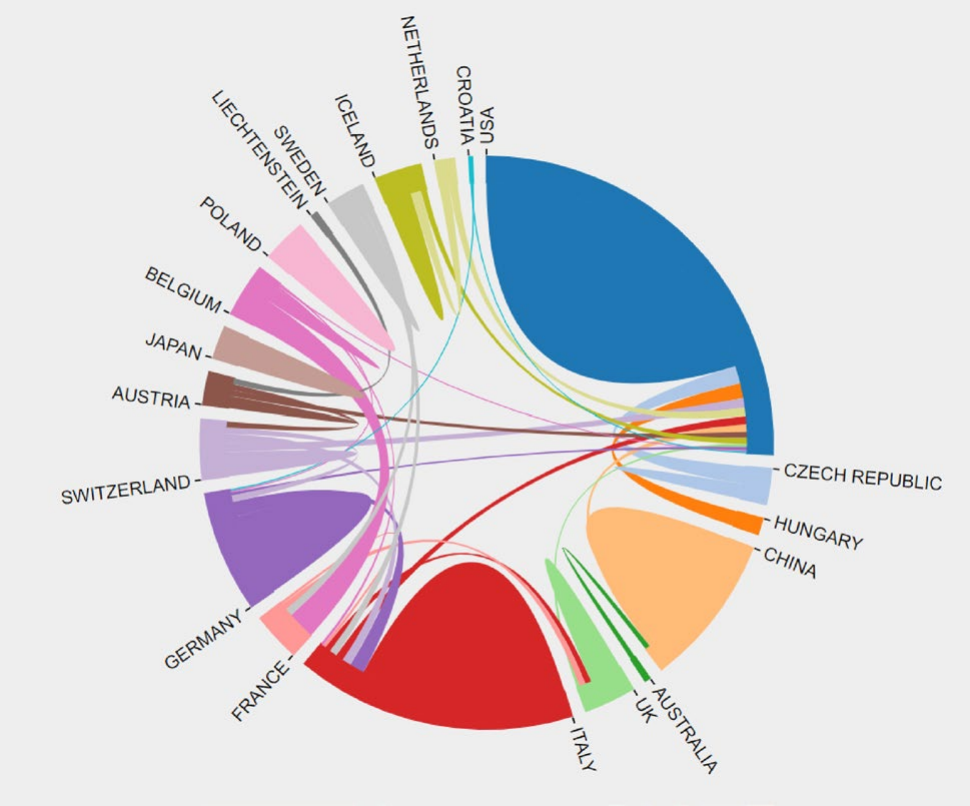
Collaboration of countries/regions involved in UMO research

A total of 353 UMOD-related papers were from 50 different countries/regions and 396 institutions. The United States published the most papers (*n* = 130), followed by Germany (*n* = 69) and Italy (*n* = 36) (Table 2). The United States had the highest centrality (centrality = 0.42), which means the United States may be a “bridge” node inumod studies (Fig. 3). The collaboration of countries/regions involved is shown in Fig. 4. To date, the United States has been the largest contributor to UMOD researches.

**Table 2:** Top 10 countries or regions publishing on UMOD

Ranking	Publications	Countries	Centrality	Countries
1	130	USA	0.42	USA
2	69	Germany	0.41	Germany
3	36	Italy	0.16	Switzerland
4	34	China	0.11	Australia
5	27	Switzerland	0.09	Italy
6	25	Austria	0.09	Austria
7	24	Netherlands	0.07	France
8	22	England	0.06	Netherlands
9	21	France	0.06	Sweden
10	19	Japan	0.06	Turkey

**Fig. 5:**
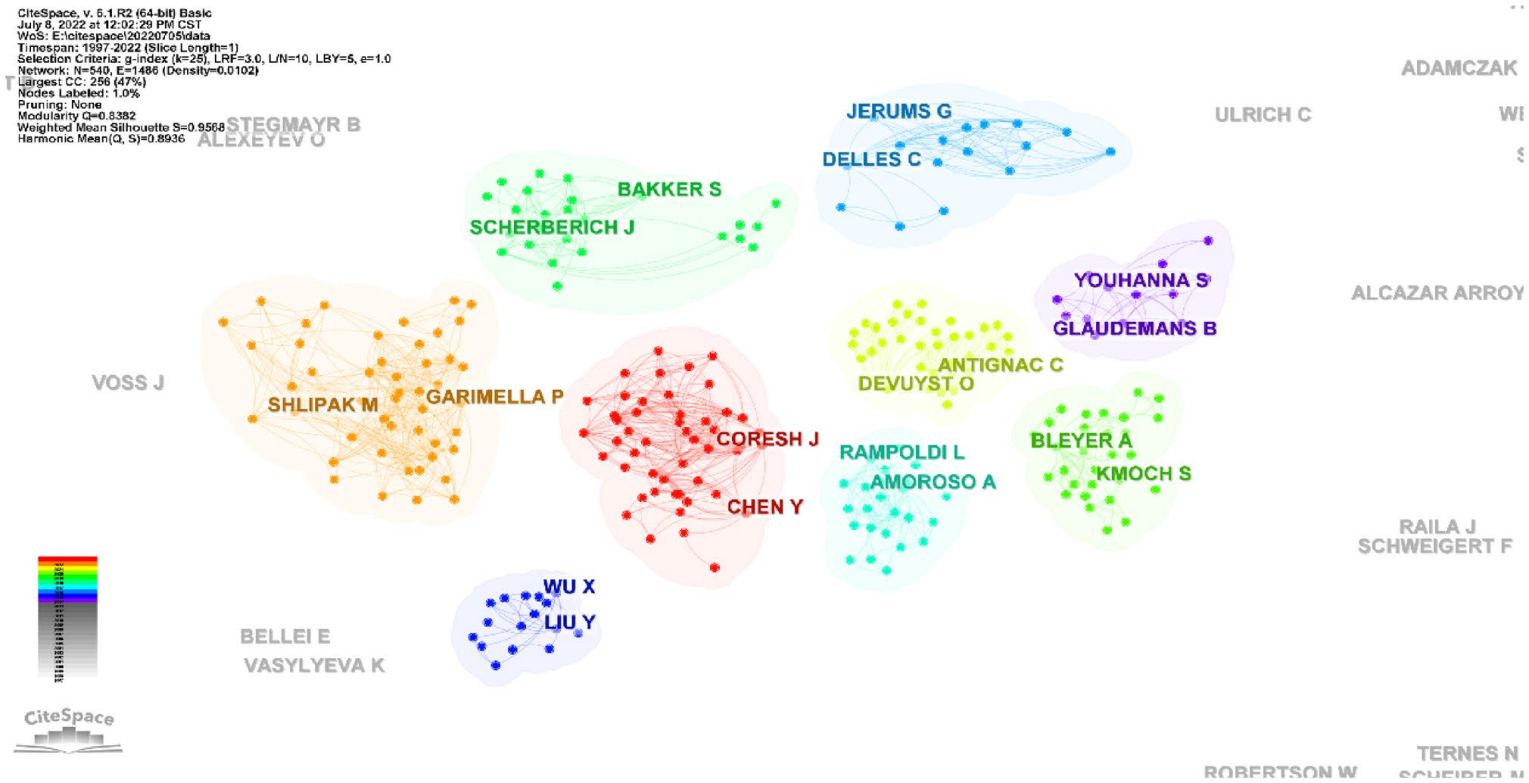
Co-occurrence map of institutions. The node size reflects the co-occurrence frequencies, and the links indicate the co-occurrence relationships. The color of node and line represent different years; colors vary from purple to red as time goes from 1997 to 2022, and nodes with purple round mean high betweenness centrality (≥ 0.1)

Univ Calif San Diego is the most productive institution (*n* = 21), followed by Univ Calif San Francisco (*n* = 17) and Charles Univ Prague (*n* = 13). However, its centrality is relatively low (*n* = 86, centrality = 0.07). By contrast, Harvard Medical School (*n* = 70, centrality = 0.14), Ghent University (*n* = 77, centrality = 0.12), and St. Jude Children’s Research Hospital (*n* = 55, centrality = 0.10) had a high centrality (Fig. 5). In addition, Brigham and Womens Hosp (centrality = 0.10), Harvard Univ (centrality = 0.08), and INSERM (centrality = 0.8) had a high centrality (Table 3).

**Table 3:** Top 10 institutions publishing on UMOD

Ranking	Publications	Institutions	Centrality	Institutions
1	21	Univ Calif San Diego	0.10	Brigham and women’s hosp
2	17	Univ Calif San Francisco	0.08	Harvard Univ
3	13	Charles Univ Prague	0.08	INSERM
4	12	Wake Forest Sch Med	0.06	Univ Calif San Francisco
5	12	Univ Zurich	0.06	Johns Hopkins Bloomber Sch Hlth
6	12	Harvard Univ	0.06	1st Sci San Raffaele
7	11	Univ Washington	0.05	Chales Univ Prague
8	11	Johns Hopkins Bloomber Sch Hlth	0.05	Univ Zurich
9	10	NYU	0.05	German Res Ctr Environm Hlth
10	10	Brigham and Women’s Hosp	0.04	Boston Univ

### Distribution of articles by authors and co-cited authors

**Fig. 6:**
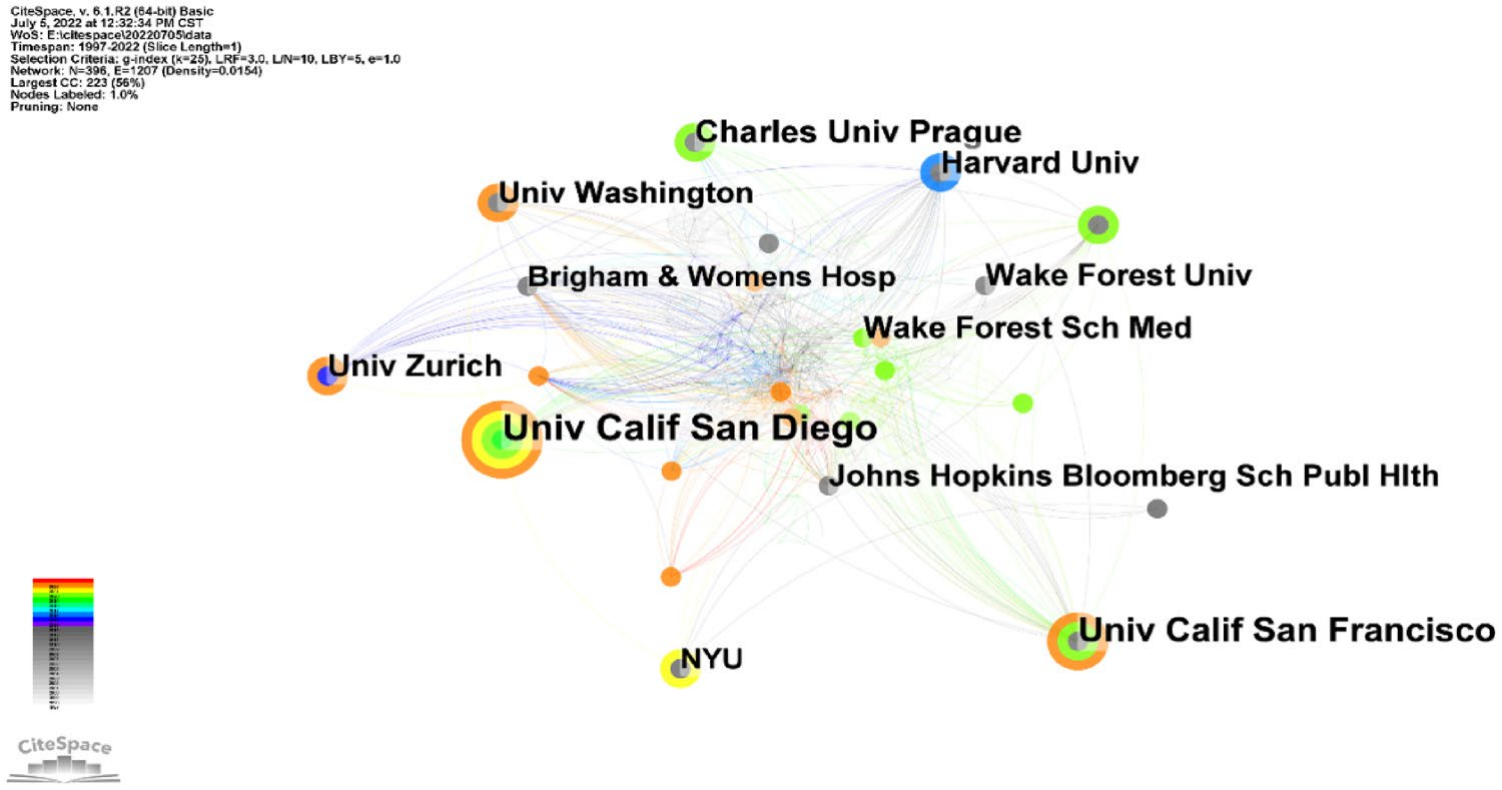
Co-occurrence authors’ map of UMOD research. The size of node reflects the authors’ co-occurrence frequencies, the link indicates the co-occurrence relationship between authors, the thickness of the link is proportional to the number of publications two researchers have co-authored, and the same color of node represents the same cluster

A total of 2346 authors were involved in the UMOD researches, among them, 91 authors published at least ten papers per person (Fig. 6; Table 4). Olivier Devuyst from the University of Zurich in Switzerland published the highest number of the UMOD-related papers (*n* = 19) followed by Anthony J Bleyer and Michael G Shlipak (Table 4). There were 9 colors in Fig. 6, representing 9 clusters among authors. Active collaborations usually exist in the same cluster, such as JERUMS G and DELLES C.

**Table 4:** Top 10 authors related to UMOD

Authors	Articles	Articles fractionalized	h_index
Devuyst O	19	2.53	13
Bleyer AJ	18	3.04	14
Shlipak MG	17	1.75	11
Ix JH	13	1.58	10
Kmoch S	12	1.77	9
Liu Y	12	1.81	9
Garimella PS	11	1.47	9
Parikh CR	11	1.43	7
Rampoldi L	11	1.61	9
Steubl D	10	0.85	7

Co-cited authors are the authors who were cited in one article [16]. Among 718 co-cited authors, 10 had over 10 co-citations (Table 5). Kottgen A is the highest frequency co-cited author (*n* = 21), followed by Rampoldi L (*n* = 17) and Hart TC (*n* = 13). However, LIU Y had the highest centrality (centrality = 0.19), followed by Hoyer JR (centrality = 0.14) and Kumar S (centrality = 0.13) (Table 5).

**Table 5:** Top 10 co-cited authors related to UMOD

Ranking	Count	Cited-author	Centrality	Cited-author
1	21	Kottgen A	0.19	Liu Y
2	17	Rampoldi L	0.14	Hoyer JR
3	13	Hart TC	0.13	Kumar S
4	12	Bleyer AJ	0.11	Bachmann S
5	12	Serafini-Cessi F	0.10	Devuyst O
6	12	El-Achkar TM	0.07	Fox CS
7	11	Levey AS	0.07	KOTTGEN A
8	11	Devuyst O	0.07	El-Achkar TM
9	10	Dahan K	0.07	Rindler MJ
10	10	Vyletal P	0.046	Cavallone D

### Journals and co-cited academic journals

A total of 193 academic journals published articles in the UMOD field. The top 10 journals published 101 papers in total, accounting for 28.61% of all publications of UMOD field (Table 6). Kidney International had the greatest number of publications (*n* = 19), followed by Nephrology Dialysis Transplantation (*n* = 17) and PLOS One (*n* = 12) (Table 6). Among 2123 co-cited sources, 31 journals had more than 100 citations, among which, SCIENCE (*n* = 170), NATURE (*n* = 164), LANCET (*n* = 160), and KIDNEY INT (*n* = 1149) had the greatest number of citations, followed by J AM SOC NEPHROL (*n* = 1135) (Table 7).

**Table 6:** Top 10 journals related to umod

Rank	Journal	Count	JCR	IF
1	Kidney International	19	Q1	18.998
2	Nephrology Dialysis Transplantation	17	Q1	7.186
3	PLOS One	12	Q2	3.752
4	American Journal of Kidney Diseases	9	Q1	11.072
5	BMC Nephrology	8	Q3	2.585
6	Clinical Journal of the American Society of Nephrology	8	Q1	10.614
7	Pediatric Nephrology	8	Q2	3.651
8	American Journal of Nephrology	7	Q1	4.605
9	Scientific Reports	7	Q3	4.996
10	Clinical Nephrology	6	Q4	1.243

**Table 7:** Top 10 co-cited journals related to umod

Rank	Journal	Count	JCR	IF
1	Kidney International	1499	Q1	18.998
2	Journal of the American Society of Nephrology	1135	Q1	14.987
3	Nephrology Dialysis Transplantation	630	Q1	7.186
4	American Journal of Kidney Diseases	529	Q1	11.072
5	American Journal of Physiology-Renal Physiology	513	Q2	4.097
6	Nature Genetics	369	Q1	41.307
7	Journal of Biological Chemistry	364	Q2	5.486
8	Clinical Journal of the American Society of NephrologY	331	Q1	10.614
9	Journal of Clinical Investigation	276	Q1	19.456
10	PLOS One	269	Q2	3.752

### Co-cited references and reference burst

Table 8 showed the top 10 of the 12,488 co-cited references. Among them, 8 of the top 10 were research articles, 2 were reviews. The top 1 of the co-cited reference is an article published in J Med Genet by T C Hart et al. in 2022 (*n* = 90). Two articles published by Luca Rampoldi from San Raffaele Scientific Institute have been cited by 59 and 56 times, respectively, which ranked to the fourth and fifth of the top 10 co-cited references [17, 18]. Three articles published by Anna Köttgen from Johns Hopkins University have been cited by 74, 51 and 45 times, respectively, which ranked to the second, seventh and tenth of the top 10 co-cited references (Table 8) [12, 19, 20].Those two authors had made great contributions to the research of UMOD, which is the foundation of the knowledge related to the UMOD research.

**Table 8:** Top 10 co-cited references related to umod

Rank	Title	First author	Type	Journal	Citation	Year
1	Mutations of the UMOD gene are responsible for medullary cystic kidney disease 2 and familial juvenile hyperuricaemic nephropathy	T C Hart	Article	J Med Genet	90	2002
2	Multiple loci associated with indices of renal function and chronic kidney disease	Anna Köttgen	Article	Nat Genet	74	2009
3	Tamm-Horsfall glycoprotein: biology and clinical relevance	Franca Serafini-Cessi	Review	Am J Kidney Dis	66	2003
4	Allelism of MCKD, FJHN and GCKD caused by impairment of uromodulin export dynamics	Luca Rampoldi	Article	Hum Mol Genet	59	2003
5	The rediscovery of uromodulin (Tamm-Horsfall protein): from tubulointerstitial nephropathy to chronic kidney disease	Luca Rampoldi	Review	Kidney Int	56	2011
6	A cluster of mutations in the UMOD gene causes familial juvenile hyperuricemic nephropathy with abnormal expression of uromodulin	Karin Dahan	Article	J Am Soc Nephrol	55	2003
7	Uromodulin levels associate with a common UMOD variant and risk for incident CKD	Anna Köttgen	Article	J Am Soc Nephrol	51	2010
8	Common noncoding UMOD gene variants induce salt-sensitive hypertension and kidney damage by increasing uromodulin expression	Matteo Trudu	Article	Nat Med	48	2013
9	Association of variants at UMOD with chronic kidney disease and kidney stones-role of age and comorbid diseases	Daniel F Gudbjartsson	Article	PLoS Genet	47	2010
10	New loci associated with kidney function and chronic kidney disease	Anna Köttgen	Article	Nat Genet	45	2010

**Fig. 7:**
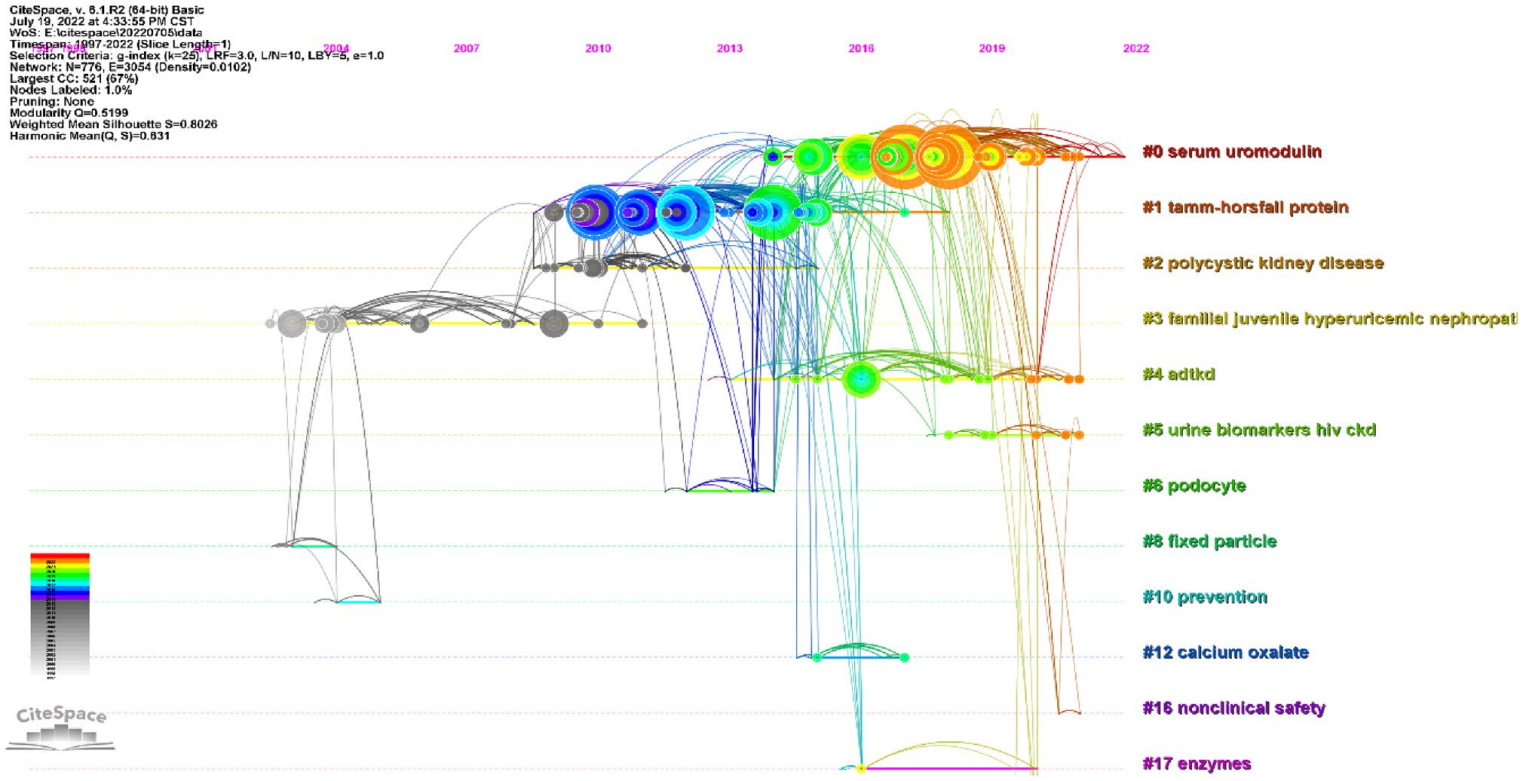
Timeline view of co-cited references related to umod. (Each horizontal line represents a cluster; the smaller the number, the larger the cluster, and #0 is the largest cluster. The node size reflects the co-cited frequencies, and the links indicate the co-cited relationships; the color of node and line represent different years; nodes are at their first co-cited year)

The references timeline view could visualize the evolution of research hotspots over time. The terms with the highest frequency in each cluster were tagged as cluster labels. As shown in Fig. 7, cluster #3(fixed particle) and #8(familial juvenile hyperuricemic nephropathy) started earlier; while cluster #0 (serum UMOD), #4 (ADTKD) and #5(urine biomarkers hiv ckd) are still ongoing, which could be considered as the frontier.

**Fig. 8:**
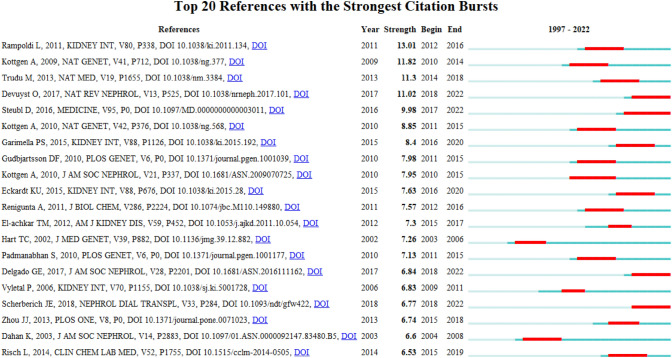
Top 20 references with the strongest citation bursts involved in umod (sorted by the strength). The Blue bars mean the reference had been published; the red bars represent citation burstness

References with citation bursts are those that have been cited with a relatively higher frequencies over a period. A total of 70 references were detected as citation bursts, and we listed the top 20 in Fig. 8. The strongest burstness (strength = 13.01) occurred in a paper entitled “The rediscovery of UMOD (Tamm–Horsfall protein): from tubulointerstitial nephropathy to chronic kidney disease”, published in Kidney Int by Rampoldi L et al. in 2011, with citation burstness from 2012 to 2016 [18]. Notably, four references were still in burstness [5, 8, 11, 21].

### Keyword analysis of trending research topic

**Fig. 9:**
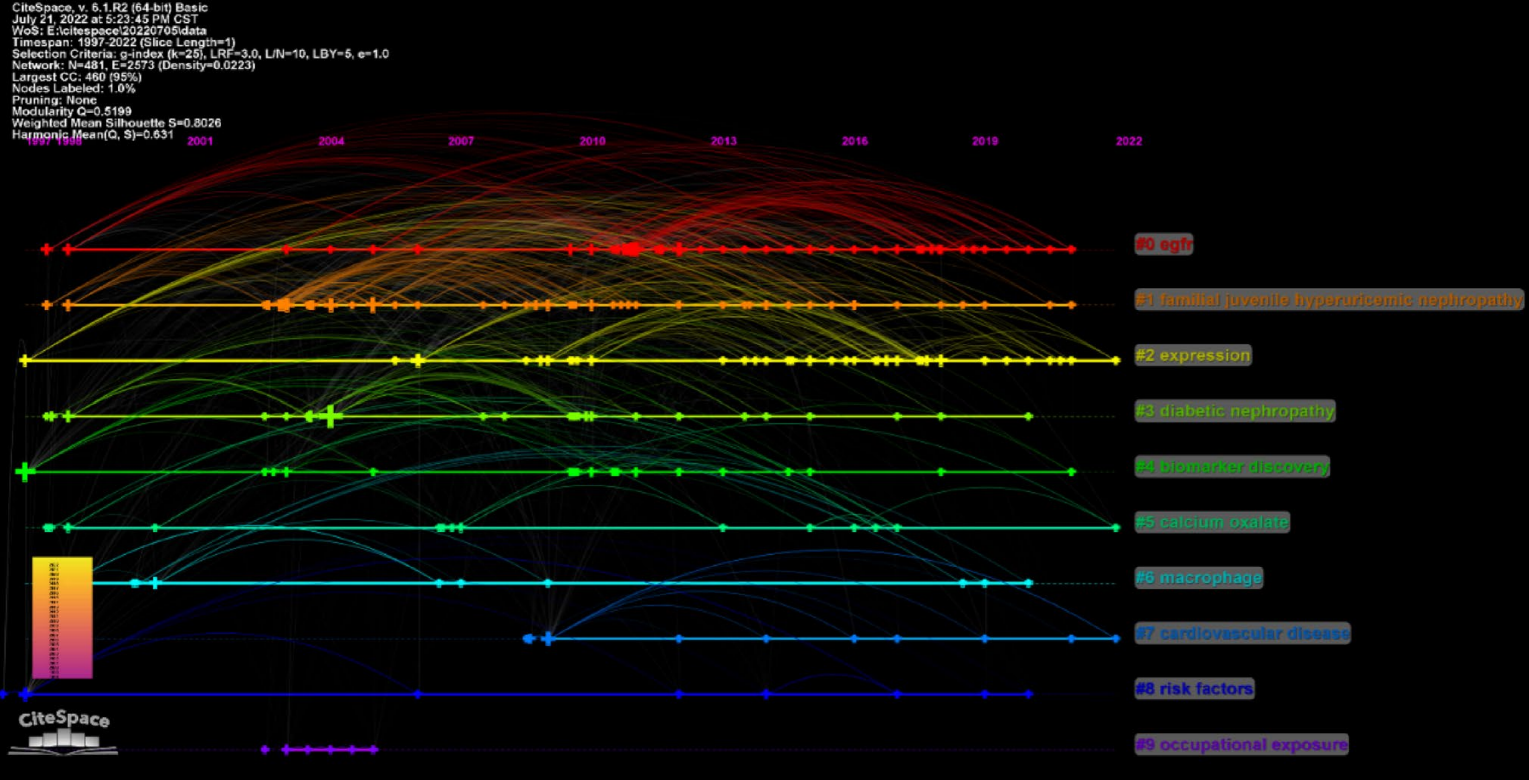
Timeline view of keywords related to umod. Each horizontal line represents a cluster; the smaller the number, the larger the cluster, and #0 is the largest. The time is at the top, and keywords are located at their first co-occurrence time in the cluster

A total of 1251 keywords were extracted, among which 48 keywords appeared at least ten times. The top 10 keywords in terms of frequency and centrality for publications in UMOD field are listed in Table 9. The most common keywords were ‘chronic kidney disease’, ‘Tamm Horsfall protein’ and ‘mutation’. Regarding the centrality index, ‘Tamm Horsfall protein’ (0.27) was the most important keyword related to UMOD, followed by ‘chronic kidney disease’ (0.25), and ‘cardiovascular disease’ (0.19). The timeline view (Fig. 9) presented the top 3 high-frequency keywords in each cluster over time. The cluster #2, #5 and #7 are still ongoing. Among them, cluster #0 (eGFR) is the biggest cluster, followed by cluster #1 (familial juvenile hyperuricemic nephropathy), cluster #2 (expression), and cluster #3 (diabetic nephropathy).

**Table 9:** Top 10 keywords related to umod

Rank	Keywords	Count	Rank	Keywords	Centrality
1	Chronic kidney disease	123	1	Tamm Horsfall protein	0.27
2	Tamm Horsfall protein	77	2	Chronic kidney disease	0.25
3	Mutation	40	3	Cardiovascular disease	0.19
4	Umod gene	37	4	expression	0.18
5	Disease	32	5	disease	0.13
6	Nephropathy	32	6	Injury	0.13
7	Expression	28	7	Activation	0.11
8	Cardiovascular disease	27	8	mutation	0.10
9	Protein	25	9	Diabetic nephropathy	0.10
10	Risk	25	10	Tamm Horsfall glycoprotein	0.09

**Fig. 10:**
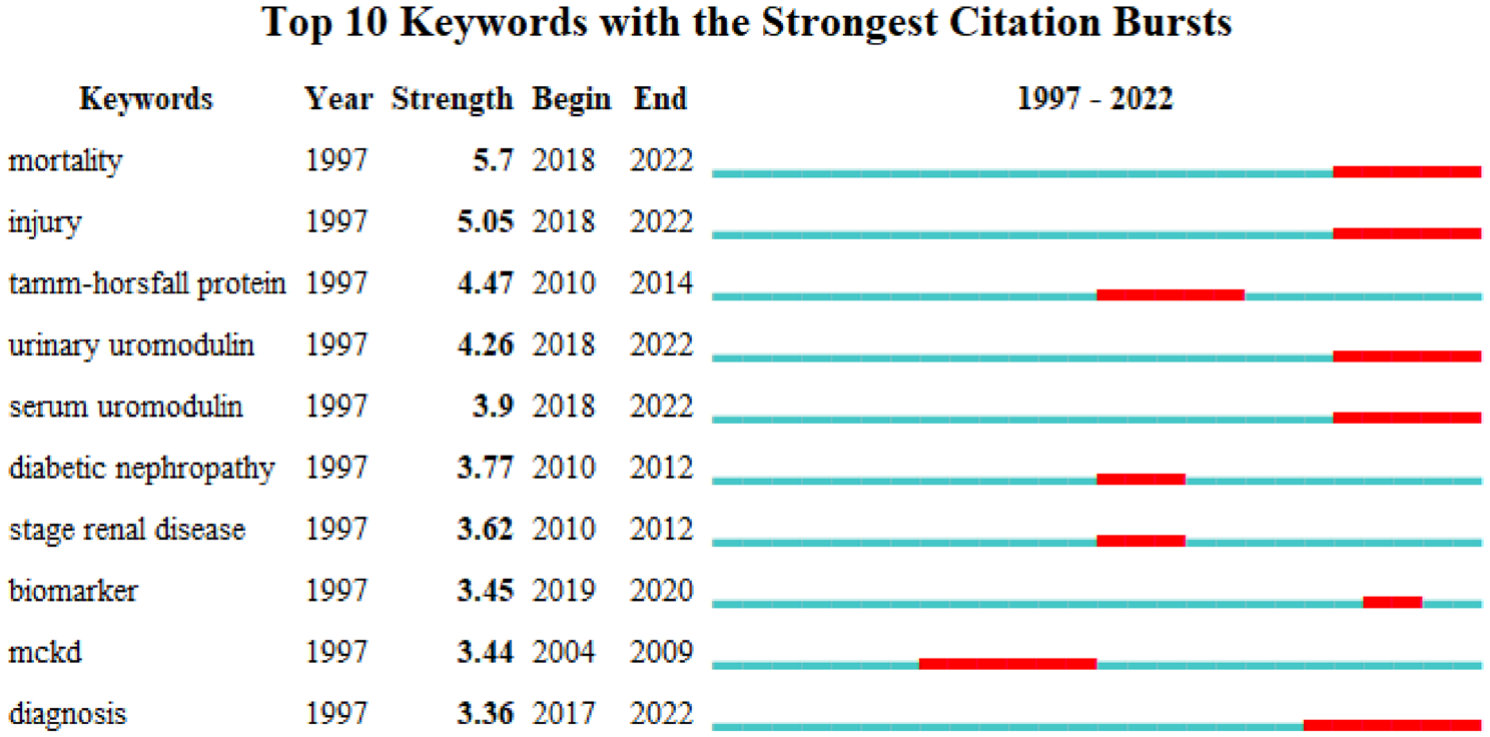
Top 10 keywords with the strongest citation bursts (sorted by strength). The red bars mean citation burstness

Keyword bursts are those that were cited significantly frequently over a period. As shown in Fig. 10, ‘mortality’ had the strongest bursts (strength = 5.7), followed by ‘injury ‘(strength = 5.05) and ‘Tamm Horsfall protein’ (strength = 8.06). Notably, ‘mortality’, ‘injury’, ‘urinary uromodulin’, ‘serum uromodulin’, and ‘diagnosis’ were still in burstness until 2022.

## Discussion

### General information

Based on the data from the WoSCC database from 1985 to 2022, a total of 353 UMOD articles were published in 193 academic journals by 2346 authors from 50 different countries/regions and 396 institutions.

The increasing trend of publications indicated that UMOD relationship with CKD is attracting mounting attention and interest. A research from the Johns Hopkins University team conducted a genome-wide association study to identify susceptibility loci for chronic kidney disease, which found that rare mutations in UMOD may influence renal function [12], since then, the UMOD research has grown rapidly.

In country/region analysis, the number of publications and betweenness centrality are two vital indicators. High centrality (≥ 0.10) nodes imply the “bridge” effects of those countries/regions in the global cooperation network [11, 22–24]. According to Table 2, Fig. 3, the United States published the most papers. In addition, of the top 10 institutions that published the most research items, 8 were from the United States according to Table 3, indicating that the United States maintain the dominant position in the UMOD research. Moreover, the United States and Germany had high centrality, which means they played key roles in the global cooperation in the UMOD field. Furthermore, in terms of network density, there was active cooperation among countries and institutions.

Among the top 10 authors and co-cited authors (Tables 4, 5), Professor Devuyst O from University of Zurich not only published the greatest number of UMOD-related papers but also ranked to the top 10 co-cited authors (count ranking is 8th, centrality ranking is 5th), indicating his outstanding contribution to the UMOD research. In 2017, his group published a review: Uromodulin: from physiology to rare and complex kidney disorders [5], which was co-cited up to 155 times and had the citation bursts from 2017 to 2022 (Fig. 8). They summarized the biochemical, physiological, genetic and pathological insights into the roles of uromodulin; the mechanisms by which UMOD mutations caused ADTKD, and the association of common UMOD variants with complex disorders.

In the journal analysis (Table 6), Kidney International published the most necroptosis studies, was also the most cited journal (Table 7). Nephrology Dialysis Transplantation and American Journal of Kidney Diseases were both the top 5 publication journals and the top 5 co-cited journals, indicating their essential role in disseminating the UMOD research.

The collection of co-cited references cited by the corresponding research community could partly represent the knowledge base [25–27]. Among the top 10 co-cited references, eight articles mainly reported the involvement of the UMOD gene in the aspectrum of rare and common kidney diseases. Two reviews summarized the UMOD investigations of biological function related to CKD. As for reference burst analysis (Fig. 8), four references are still in burst and worth our attention: one observation study reported that plasma UMOD serves as a robust biomarker for kidney function and uniquely allows the identification of early stages of CKD [28]. One of the prospective cohort studies reported that serum UMOD may be a useful marker of cardiovascular and renal health [21]. The other concluded that serum UMOD is a novel sensitive kidney-specific biomarker linked to the structural integrity of the distal nephron and to the renal function [29].One reviews summarized biochemical, physiological, genetic and pathological insights into the roles of UMOD [5].

### The hotspots and trending

It’s critical to researchers to keep abreast of the trending in the research field in such an era of information explosion. Bibliometrics provides a method in which keyword co-occurrence can reflect the hotpots of an academic area [30]. The timeline view can present the evolution of new hotpots [9, 31], and reference clusters and citation bursts can characterize the emerging topics in the discipline [23, 26, 32].In this study, we tried to objectively evaluate the hotpots and frontiers of UMOD research through the analysis of keyword co-occurrence (Table 9), keyword timeline (Fig. 9), keyword burst (Fig. 10), reference timeline (Fig. 7), and reference burst (Fig. 8). We summarized the aspects as follows.

### Biological relevance of the UMOD to kidney function

More conclusive evidence was obtained in vivo through the characterization of UMOD gene knockout mice. The UMOD polymers promotes clearance of the pathogens by forming a 3D meshwork acting as a “fishing net” that can trap microorganisms to eliminate them by micturition [33, 34], which was later confirmed in vivo [35].The protein in the urine protect are against kidney stone formation [36]. Consistent results were observed in several case–control studies showing decreased urinary UMOD levels in kidney stone formers [37–39]. UMOD was shown in vitro to decrease caveolin-mediated endocytosis of TRPV5 and TRPV6 (two channels responsible for transcellular Ca2+ reabsorption), which increased surface abundance of both channels and thus increased calcium reabsorption [40, 41]. UMOD plays an important role in sodium homeostasis in the TAL and DCT, thus regulating blood pressure and urinary concentrating ability [42]. In vivo studies ischemia–reperfusion injury models of acute kidney injury (AKI) showed increased inflammation and necrosis in kidneys of UMOD gene knockout mice, suggesting a protective role of UMOD in kidney [43]. All these processes are relevant when considering the onset of CKD, tubulointerstitial injury, and the complications arising from kidney failure.

A transgenic mouse model overexpressing wild-type UMOD, displayed a dose-dependent increase in systolic blood pressure, with salt-sensitive hypertension. Aging kidneys from the UMOD transgenic mice showed focal lesions (e.g., tubular casts, cysts) and increased expression of kidney damage markers (e.g., lipocalin-2, Kim-1) and chemokines. Genetically driven higher production of uromodulin becomes deleterious over time, promoting the onset of CKD [44].In some genome-wide association studies(GWAS), the UMOD alleles associated with higher uromodulin expression/levels are associated with increased risk of CKD, hypertension, and hyperuricemia, whereas they are protective against kidney stones [38, 45],indicating it’s importance for a set of common diseases.

### Rare mutations in UMOD

‘Familial juvenile hyperuricemic nephropathy’ (FJHN) was mentioned in Figs. 7 and , ‘Autosomal dominant tubulointerstitial kidney disease’(ADTKD) in Fig. 7. These diseases including ‘familial juvenile hyperuricemic nephropathy (FJHN)’, ‘medullary cystic kidney disease (MCKD) type 2’, ‘uromodulin-associated kidney disease (UAKD)’, UMOD-related diseases, and ‘MCKD type 1’, suggested the adoption of new terminology using a single name ‘autosomal dominant tubulointerstitial kidney disease’ (ADTKD) by KDIGO [46]. Rare dominant mutations in UMOD represent the most frequent cause of ADTKD (ADTKD–UMOD).

ADTKD is an increasingly recognized cause of end-stage kidney disease, characterized by tubular damage and interstitial fibrosis of the kidney in the absence of glomerular lesions. Affected individuals present with urinary concentrating defects, progressive CKD, normal-to-mild proteinuria, and normal-sized kidneys, often has a positive family history [47, 48]. Studies of mouse models carrying uromodulin mutations confirmed that intracellular accumulation of mutant uromodulin may lead to ER stress, induction of the unfolded protein response, and subsequent tubular damage and interstitial fibrosis—substantiating the gain of toxic function mechanism in ADTKD–UMOD [45]. In addition, the defective biogenesis and intracellular uromodulin transport affect the Na–K–2Cl cotransporter resulting in defective urinary concentration and mild volume depletion, secondarily increasing proximal reabsorption of uric acid, leading to hyperuricemia [18].

### Potential applications of UMOD in CKD

According to our results (Figs. 7, 9, 10), ‘biomarker’ is all mentioned. ‘mortality’, ‘injury’, ‘urinary uromodulin’, ‘serum uromodulin’, and ‘diagnosis’ were in burstness until 2022. In both SKIPOGH and CoLaus cohorts studies, positive associations were found between uromodulin and urinary sodium, chloride, and potassium excretion and osmolality [49]. In SKIPOGH, 24-h uromodulin excretion was positively associated with kidney length and volume and with creatinine excretion and urine volume. However, it was negatively associated with age and diabetes. The associations of UMOD excretion with markers of tubular functions and kidney dimensions suggest that it may reflect the distal tubular transport activity (e.g., reabsorption of NaCl and/or divalent cations) in the general population. In CoLaus, the data substantiate the measurement of urinary UMOD levels as a useful surrogate marker for nephron mass. As such, higher UMOD levels may indicate a higher functional reserve of the kidney with a lower risk of acute kidney injury (AKI) [38, 45]. This situation should not be confused with the results of the GWAS studies indicating that the risk of variants of the UMOD locus, which drive higher production of uromodulin reflected by higher levels in urine and blood, are consistently associated with an increased risk of CKD) [45]. Taken together, these studies validated urinary uromodulin as a biomarker for tubular mass and tubular function in the general population as well as in the disease subgroups.

The association of UMOD with rare disorders and more common conditions, as well as the availability of validated ELISA methods for measuring urinary and serum uromodulin levels support the use of uromodulin as a biomarker of tubular function in healthy individuals and in patients with kidney disease. Early studies based on radioimmunoassays found reduced uromodulin excretion in patients with tubular damage or autosomal dominant polycystic kidney disease (ADPKD) [5]. Studies based on mass spectrometry or ELISA identified uromodulin as a potential biomarker in Fabry nephropathy, active lupus nephritis with tubulointerstitial inflammation and in ADPKD [50–52]. Discovering of UMOD will drive multi-level studies substantiating cellular mechanisms and possible drug targets.

## Strength and limitations

Overall, this is the first bibliometric study to systematically analyze the relationship between UMOD and CKD. Compared to traditional reviews, the bibliometric analysis provides this novel and objective insight into the evolving research foci and trends [10]. Meanwhile, we used various bibliometric software to perform an analysis, which could provide richer results in multiple dimensions [53].This study will inform the scholars of the importance of UMOD, and serve as a comprehensive and objective guide for the future development of the UMOD research field. Inevitably, this study has some limitations. First, we exclusively retrieved the articles published in English from the WoSCC database, thus omitting articles that are not in WoSCC or not in English. Nevertheless, we are certain that the WoSCC provides greater transparency with regard to the coverage than other resources. Second, bibliometric methods also introduce inherent bias in our analysis, which based on natural language processing. Third, compared with older articles, recent articles did not have a tendency to accrue more citations. This may lead to several high-quality articles being excluded from our analysis.

## Conclusions and perspectives

In the past two decades, the scientific interest in UMOD has grown significantly with active cooperation worldwide. United States might maintain the dominant position in UMOD research. Olivier Devuyst published the highest number of UMOD-related papers. The present studies suggested that UMOD might regulate salt transport, protect against urinary tract infection and kidney stones, and have roles in kidney injury and innate immunity. UMOD has been recognized as the largest effect on eGFR and CKD incidence and progression. Furthermore, the urinary and serum level of UMOD were shown to have a prognostic value for kidney function decline and the incidence of CKD, AKI, and ESRD. In addition, UMOD was also shown to be an effective biomarker for tubular function and nephron mass, possibly an alternative to current markers that are based on glomerular function. The molecular mechanisms linking UMOD expression and CKD risk are still unclear. The pathways through which UMOD regulates the function of ion transporters need to be further dissected. All these represent areas of great interest for future research on this multifaceted protein. It is still unknown whether there are differences in the post translational modifications of urinary and serum UMOD other than polymerization that may be associated with different biological functions.

## Data Availability

The original contributions presented in the study are included in the article/Supplementary Material. Further inquiries can be directed to the corresponding authors.
